# Host Innate Immunity Against Hepatitis Viruses and Viral Immune Evasion

**DOI:** 10.3389/fmicb.2021.740464

**Published:** 2021-11-03

**Authors:** Chonghui Xu, Jizheng Chen, Xinwen Chen

**Affiliations:** ^1^State Key Laboratory of Virology, Wuhan Institute of Virology, Center for Biosafety Mega-Science, Chinese Academy of Sciences, Wuhan, China; ^2^University of Chinese Academy of Sciences, Beijing, China; ^3^Guangzhou Institutes of Biomedicine and Health, Chinese Academy of Sciences, Guangzhou, China

**Keywords:** hepatitis virus, viral infection, antiviral innate immunity, immune evasion, replication

## Abstract

Hepatitis viruses are primary causative agents of hepatitis and represent a major source of public health problems in the world. The host innate immune system forms the first line of defense against hepatitis viruses. Hepatitis viruses are sensed by specific pathogen recognition receptors (PRRs) that subsequently trigger the innate immune response and interferon (IFN) production. However, hepatitis viruses evade host immune surveillance via multiple strategies, which help compromise the innate immune response and create a favorable environment for viral replication. Therefore, this article reviews published findings regarding host innate immune sensing and response against hepatitis viruses. Furthermore, we also focus on how hepatitis viruses abrogate the antiviral effects of the host innate immune system.

## Introduction

Hepatitis is an inflammation of the liver that gives rise to a wide spectrum of systemic manifestations and can cause death. It continues to be a global human health threat and economic burden. According to the World Health Organization (WHO), about 325 million people suffer from chronic hepatitis infection. There are five main strains of the hepatitis virus [type A, B, C, D, and E] (Carman and Thomas, 1990; Purcell, 1993)—all of which cause liver disease, but differ in some aspects, such as mode of transmission, severity of illness, geographical distribution, and prevention methods (Acorn et al., 1995; Rasche et al., 2019). Hepatitis A virus (HAV) and hepatitis E virus (HEV) are responsible for a large proportion of cases of acute hepatitis, while hepatitis B virus (HBV) and hepatitis C virus (HCV) are the most common causes of chronic viral hepatitis. Hepatitis D virus (HDV) is a satellite virus that requires the envelope proteins of HBV for cell release and uptake (Hughes et al., 2011; Tu et al., 2017; Smith and Simmonds, 2018).

The host innate immune response is the first line of defense against virus invasion. During virus infection, the pathogen-associated molecular patterns (PAMPs) of the virus are recognized by host pathogen recognition receptors (PRRs), which is the first step of antiviral innate immunity activation (Lester and Li, 2014; Streicher and Jouvenet, 2019). Viral nucleic acid, which represents a major PAMP, can be recognized by various PRRs, including retinoic acid-inducible gene-I (RIG-I)-like receptors (RLRs), Toll-like receptors (TLRs), and nucleotide-binding oligomerization domain (NOD)-like receptors (NLRs), as well as cytosolic DNA sensors, such as cyclic GMP-AMP (cGAMP) synthase (cGAS), interferon (IFN) regulatory factors (DAIs), and interferon gamma inducible protein 16 (IFI16). PRRs then recruit downstream adaptor proteins, which typically include mitochondrial antiviral-signaling protein (MAVS), TIR-domain-containing adaptor-inducing IFN-β (TRIF), myeloid differentiation primary response gene 88 (MyD88), and intracellular stimulator of IFN genes (STING). These adaptor proteins ultimately lead to the activation of the transcription factor nuclear factor-kappa B (NF-κB) and interferon regulatory factor 3 (IRF3), subsequently inducing the expression of type I IFNs and pro-inflammatory cytokines, which contribute to the establishment of the “antiviral state” (Koyama et al., 2008; Wan et al., 2020; Carty et al., 2021).

As a key, frontline immune tissue, the liver is able to mount a rapid and robust immune response under appropriate conditions. It is enriched with innate immune cells, including Kupffer cells (KCs), natural killer (NK) cells, natural killer T (NKT) cells, and hepatic dendritic cells [DCs] (Gao, 2016; Ringelhan et al., 2018). Evidence suggests that hepatocytes are responsible for the biosynthesis of 80–90% of innate immune proteins, including complement components and secreted PRRs, and the liver can express membrane-bound PRRs, such as TLRs (Gao et al., 2008, 2011). However, viruses have also evolved a variety of strategies to evade the innate immune response in order to optimize their replication capacity. Research regarding hepatitis viruses, in particular HBV and HCV, has identified several molecular mechanisms hepatitis viruses employ to evade the host innate immune system (Foy et al., 2003; Johnson et al., 2007; Pugnale et al., 2009; Dong et al., 2012; Hong et al., 2015; Liu et al., 2015; Yi et al., 2015; Feng and Lemon, 2019; Wang et al., 2019). Here, we provide an overview of how hepatitis viruses trigger and evade the host antiviral innate immune response.

## Hepatitis A Virus

**FIGURE 1:**
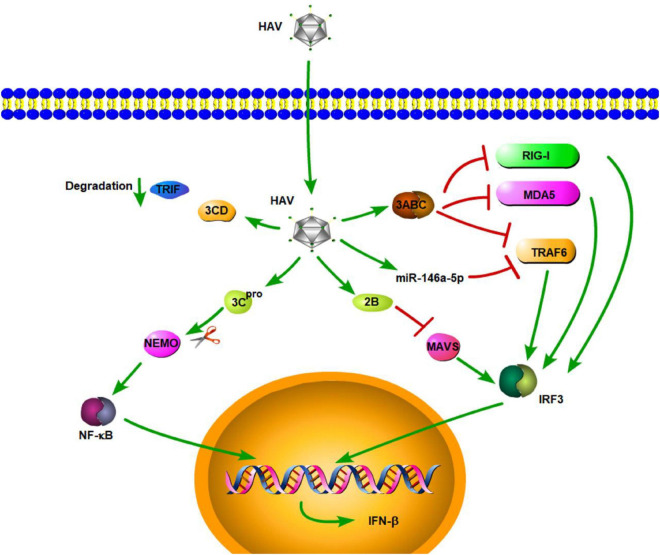
Hepatitis A virus suppression of type I interferon (IFN) response (green arrows indicate the related signaling pathways and T-arrows in red indicate blocking the targets). HAV 3ABC downregulates MAVS to disrupt the RIG-I and MDA5 pathways, thereby inhibiting the type I IFN response. HAV increased the expression of hsa-miR-146a-5p, thus leading to the cleavage of TRAF6.HAV 3CD can induce TRIF degradation. HAV 3C^pro^ cleaves nuclear factor-κB essential modulator (NEMO), the regulatory subunit for IκB kinase (IKK) that controls the NF-κB signaling pathway. HAV 2B interferes with the activity of MAVS and affects the function of IRF3 kinases.

Hepatitis A virus is a small, hepatotropic, positive-strand RNA virus transmitted by the fecal–oral route. In contrast to HBV and HCV, which cause chronic hepatitis, HAV generally causes only acute disease, and patients may develop multiple symptoms of acute hepatitis, such as acute liver failure [ALF] (Shin and Jeong, 2018). To date, the innate immune response to acute HAV infection has been reported, but not carefully examined. Previous studies have shown that TLR3, RIG-I, and melanoma differentiation-associated gene-5 (MDA5) are the main PRRs involved in HAV recognition, which leads to the induction of an antiviral state (Fensterl et al., 2005; Yang et al., 2007; Qu et al., 2011). Compared with acute resolving HCV infection, there is a very limited induction of type I IFN-stimulated genes in HAV-infected animals (Lanford et al., 2011). This similar result is also found in the study by Brack et al. (2002). They demonstrated that HAV can inhibit double-stranded RNA (dsRNA)-induced IFN-β gene expression (Brack et al., 2002). These results indicated that HAV is stealthier than HCV in acute resolving infection. Consistent with the conclusions of that study, some studies have shown that HAV can actively employ strategies to evade the innate immune response. These strategies, to some degree, contribute to the stealth of HAV. MAVS is a central adaptor protein that plays a critical role in RIG-I- and MDA5-mediated signaling. The activation of MAVS leads to the induction of type I IFNs through the activation of transcription NF-κB and IRF3. Yang et al. (2007) showed that downregulation of MAVS by HAV 3ABC disrupts the RIG-I and MDA5 pathways, thereby inhibiting the type I IFN response (Figure 1). They also found that HAV disrupts TLR3 signaling by targeting the essential adaptor protein, TRIF. Moreover, the degradation of TRIF was shown to be caused by HAV 3CD (Qu et al., 2011). HAV can disrupt RIG-I, MDA5, and TLR3 signaling pathways through cleavage of essential adaptor proteins by two distinct protease precursors derived from the common 3ABCD polyprotein processing intermediate (Figure 1). TNF receptor-associated factor 6 (TRAF6) is also a key adaptor protein in RIG-I- and MDA5-mediated type I IFN signaling pathways. A recent study by Mo et al. (2021) demonstrated that, aside from MAVS and TRIF, HAV also disrupts IFN-β signaling, in part, through the cleavage of TRAF6 mRNA via microRNAs (miRNAs). They found HAV can increase the expression of hsa-miR-146a-5p, thus leading to the cleavage of TRAF6 (Figure 1; Mo et al., 2021). Furthermore, HAV 3C^pro^ cleaves nuclear factor-κB essential modulator (NEMO), the regulatory subunit for IκB kinase (IKK) that controls the NF-κB signaling pathway, at the Q304 residue, which negates the signaling adaptor function of NEMO, thereby abrogating RIG-I, MDA5, and TLR3 pathways (Figure 1; Wang et al., 2014). In addition to HAV 3ABC, another HAV protein, 2B, also has an inhibitory effect on MAVS-mediated IRF3 activation. HAV 2B interferes with the activity of MAVS and affects the function of IRF3 kinases, TANK-binding kinase 1 (TBK1), and the inhibitor of NF-κB kinase ε [IKKε] (Paulmann et al., 2008). Furthermore, experiments in a mouse model of HAV infection have also indicated that HAV can also evade the MAVS-mediated type I IFN response (Hirai-Yuki et al., 2016). According to the above results and existing literature, cleavage of important adaptor proteins is the primary way for HAV to antagonize the innate immune response. In addition, studies have also shown that plasmacytoid dendritic cells (pDCs) can rapidly produce a large amount of type I IFNs in response to foreign nucleic acids, and this function of pDCs is connected with TLR7 and TLR9 (Gilliet et al., 2008; Reizis et al., 2011). As for HAV, pDCs produce large amounts of IFN-α via TLR7 signaling when co-cultured with HAV-infected cells. Interestingly, while host membrane envelopment has been shown to protect HAV from host neutralizing antibodies, it also contributes to the early detection of HAV by pDCs (Feng et al., 2015).

## Hepatitis B Virus

Hepatitis B virus is a partially double-stranded DNA virus belonging to the *Hepadnaviridae* family. HBV infection remains the most common chronic viral infection in the world. If left untreated, chronic HBV infection progress to end-stage liver disease, such as liver cirrhosis and hepatocellular carcinoma [HCC] (Li H. et al., 2020). During infection, HBV has the ability to stimulate the innate immune response. According to published studies, HBV triggers the innate immune system, but it also has the capability of using a variety of powerful strategies to evade the innate immune response and induce immunosuppression.

### Toll-Like Receptors

Toll-like receptors play significant roles against HBV infection. TLRs, including TLR3, TLR4, TLR5, TLR7, and TLR9, can inhibit HBV replication in an IFN-α/β-dependent manner in HBV transgenic mice within 24 h (Isogawa et al., 2005). HBV RNA is produced in infected cells, and dsRNA is the main target for TLR3. Chen et al. (2015) reported that the expression of TLR3 in human HBV-associated HCC tissues is related to HBV infection. The upregulation of the synthesis of dsRNA in HCC with HBV infection can activate TLR3, which in turn, promotes interstitial immunoreactive cells and induces production of inflammatory cytokines (Chen et al., 2015). As for TLR4, a previous study showed that TLR4 activation represses HBV infection through the NF-κB pathway (Das et al., 2016; Jing et al., 2018). Another study also revealed that peripheral blood mononuclear cells (PBMCs) isolated from chronic HBV (CHB)-infected patients have decreased expression of TRIF, which may attenuate TLR3 and TLR4 signaling subsequent to HBV infection (Ayoobi et al., 2013) and might be one of the reasons why HBV infection is stable in CHB patients. Numerous studies have reported various TLR7 agonists, which have been used as adjuvants to induce immune responses, to have diverse effects against HBV (Sepehri et al., 2016), including R-848 (a ligand for TLR7), which promotes an immune response against HBsAg (Ma et al., 2007). Xu et al. reported that PBMCs from CHB patients showed reduced production of IFN-α in response to Loxoribine [a ligand for TLR7] (Xu et al., 2012). These results suggested that TLR7 plays a key role in the induction of an immune response against HBV. Another study also revealed that TLR9 can recognize HBV DNA, resulting in activation of immune cells (Sajadi et al., 2013; Shahrakyvahed et al., 2014). Furthermore, a recent study by Zhang et al. (2020) found that TLR2 is involved in the recognition of HBV during the early infection process and initiates innate immune signaling in primary human hepatocytes (PHHs), demonstrating that TLR2 is an important TLR in the fight against HBV. Additionally, several TLRs, including TLR3 (An et al., 2007; Li et al., 2009), TLR4 (Chen et al., 2008), TLR7 (Xu et al., 2012; Lin et al., 2013), and TLR9 (Vincent et al., 2011; Sajadi et al., 2013; Shahrakyvahed et al., 2014), are downregulated in CHB patients. Thus, downregulating TLRs might be a strategy for HBV to evade host TLR-activated innate immune responses.

**FIGURE 2:**
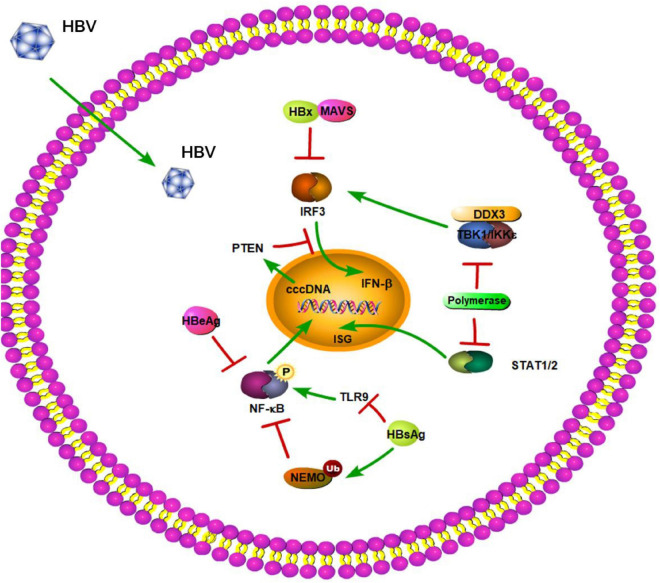
Hepatitis B virus suppression of type I interferon (IFN) response (green arrows indicate the related signaling pathways and T-arrows in red indicate blocking the targets). HBsAg inhibits TLR9 and mediates the ubiquitination of NEMO to suppress the NF-kB pathway. HBeAg can inhibit phosphorylation of NF-kB. HBV polymerase can block the nuclear translocation of activator of transcription 1 (STAT1). HBV polymerase is able to disrupt the interaction between TBK1/IKKε and DEAD box protein 3 (DDX3) to play a novel role in HBV counteraction of IFN-β production. HBx interacts with MAVS to act as a suppressor of virus-triggered induction of type I IFNs. HBV infection can also reduce PTEN protein expression to inhibit IFN signaling pathway.

Moreover, studies have shown that HBV surface antigen (HBsAg), HBV e antigen (HBeAg), and even HBV virions can suppress TLR-mediated innate immunity and IFN-β induction in liver cells (Figure 2; Wu et al., 2009). A previous study demonstrated that HBsAg inhibits TLR9-mediated IRF7 expression and nuclear translocation, which are important for induction of IFN-α gene transcription (Figure 2; Xu et al., 2009). Furthermore, HBeAg can associate with NEMO and inhibit TRAF6-mediated K63-linked ubiquitination of NEMO (Figure 2). The decreased ubiquitination of NEMO contributes to the downregulation of NF-κB activity, thus promoting the replication of HBV (Wang et al., 2019). Besides regulating the modification of NEMO, HBeAg protein also can modulate the NF-κB pathway in hepatoma cells via suppressing IL-18 and IL-1β-mediated NF-κB signaling (Wilson et al., 2011; Jegaskanda et al., 2014). Lang et al. provided a molecular mechanism describing HBeAg immunomodulation of the innate immune signal transduction pathways. They found that HBeAg, but not the HBV core antigen, co-localizes with Toll/IL-1receptor (TIR)-containing proteins TRAM and Mal at the plasma membrane. HBeAg can then suppress TIR-mediated activation of the inflammatory transcription factor NF-κB and IFN-β promoter activity and disrupt homotypic TIR:TIR interaction, which is critical for TLR-mediated signaling (Lang et al., 2011). Additionally, a previous study demonstrated that after culturing PBMCs with various concentrations of HBeAg for 72 h, HBeAg can inhibit proliferation of lymphocytes, increase TLR3, TLR4, and PD-1 expression, and decrease IFN-γ production (Chen et al., 2017). The inhibition of IFN-γ expression is also found in HBeAg-positive rather than HBeAg-negative CHB patients (Jegaskanda et al., 2014). MyD88 is induced by IFN-α and acts as a critical component in the signaling cascade through TLRs. Xiong et al. (2004) found that the levels of HBV proteins and viral replicative intermediates are effectively reduced in MyD88-expressing cells, indicating that MyD88 has antiviral activity against HBV replication. Based on this, Wu et al. (2007) reported that HBV polymerase can inhibit expression of the IFN-α-inducible MyD88 by inhibiting the activity of the MyD88 promoter, possibly by blocking the nuclear translocation of activator of transcription 1 [STAT1] (Figure 2). In addition, transcriptional activity of the IFN-stimulated response element (ISRE) promoter and expression of interferon-stimulated genes (ISGs) are also downregulated by HBV polymerase (Figure 2; Wu et al., 2007). In a certain respect, these results indicate that HBV viral proteins play important roles against TLR-mediated innate immune responses.

### RIG-I-Like Receptors

RIG-I-like receptors are key PRRs that detect virus-derived RNAs. A recent clinical study not only showed that RLRs have an effect on HBV clearance in the Chinese Han population, but also identified three single-nucleotide polymorphisms (SNPs; *DDX58* rs3824456, *DHX58* s2074160, and *IFIH1* rs2111485) in RLRs that may be associated with spontaneous clearance of HBV (Yao et al., 2021). Lee et al. (2021) also revealed that activation of the RIG-I pathway offers therapeutic benefits toward elimination of HBV covalently closed circular DNA [cccDNA]. Different HBV genotypes can be recognized by diverse RLRs. In a previous study, Sato et al. (2015) reported that RIG-I can sense HBV genotype A, B, and C and recognize the 5′-ε region of HBV pgRNA. Furthermore, RIG-I dampens HBV replication through counteracting the interaction between HBV polymerase and pgRNA as well (Sato et al., 2015). In turn, HBV polymerase was shown to be able to disrupt the interaction between TBK1/IKKε and DEAD box protein 3 (DDX3) to play a novel role in HBV counteraction of IFN-β production (Yu et al., 2010). Recently, a further study by Zhou et al. also revealed that HBV impedes RIG-I-induced IFN production through sequestering MAVS from RIG-I by forming a ternary complex including hexokinase (HK). This study provided a novel mechanism of HBV immune escape in which HBV promotes glucose metabolism and suppresses RIG-I-MAVS signaling (Zhou et al., 2021). Another RLR, MDA5, can sense the genotype D of HBV and associate with HBV DNA (Lu and Liao, 2013). However, it is unknown whether the association of MDA5 with DNA is required for MDA5-mediated signaling. Interestingly, across-sectional study showed that mRNA levels of MDA5 and RIG-1 are significantly decreased and increased, respectively, in CHB patients when compared to healthy controls (Ebrahim et al., 2015). It is easy to understand that decreased expression of MDA5 results in impaired HBV recognition and consequently disrupts the immune response in CHB patients. However, it is confusing that RIG-I plays an opposing role in CHB patients. Subsequently, Zeng et al. (2019) revealed that the levels of RIG-I are significantly decreased in CHB patients in the active phase when compared to healthy controls, which is contradictory to the results of Ebrahim et al. (2015). This suggested that the expression level of RIG-I might be related to the progression of CHB infection. MAVS is a key adaptor protein in RIG-I- and MDA5-mediated signaling. Downregulation of MAVS-induced signaling by HBV-encoded X protein (HBx) has already been proposed (Figure 2). A study by Kumar et al. (2011) identified MAVS as one of the novel cytoplasmic binding partners of HBx, which specifically inhibits the double-stranded DNA (dsDNA) pathway of IFN-β activation in a dose-dependent manner. Wang et al. (2010) also found HBx interacts with MAVS and acts as a suppressor of virus-triggered induction of type I IFNs by disrupting the association of MAVS with its upstream and downstream components (Figure 2). Moreover, HBx promotes the degradation of MAVS, thereby attenuating the antiviral response of the innate immune system (Figure 2). Further analysis of clinical samples also revealed that MAVS protein is downregulated in HBV-induced HCC (Wei et al., 2010). These results provide possible explanations into how HBV is able to avoid activating MAVS-induced innate immune responses during virus infection. Apart from affecting key innate immune adaptor proteins, the roles of m^6^A in RNAs are emerging in viral immune regulation. Kim et al. (2020) demonstrated that m^6^A modification of HBV pgRNA and HCV genomic RNA promotes their interactions with YTHDF proteins, and thereby, disrupts RIG-I sensing activity. Recently, they also revealed that HBV can affect tumor suppression, including phosphatase and tensin homolog (PTEN), which activates IFN signaling pathway by dephosphorylation of IRF3 at Ser97 (Figure 2). HBV infection can also reduce PTEN protein expression by increasing the m^6^A methylation of PTEN mRNA (Kim et al., 2021). The elimination of PTEN results in the inhibition of p-IRF3 nuclear import. Kim et al. (2020, 2021) provided new insights into the mechanism of immune evasion via m^6^A modification of RNAs.

### NOD-Like Receptors and STING-Dependent DNA Sensor

Currently, the NLR family pyrin domain-containing 3 (NLRP3) inflammasome is the most studied inflammasome. Yu et al. (2017) found that HBeAg, but not HBsAg, suppresses LPS-induced NLRP3 inflammasome activation and IL-1β production via inhibiting NF-κB phosphorylation and ROS production. This inhibitory effect of HBeAg has been confirmed in CHB patients and might contribute to the HBV persistence and immune tolerance (Yu et al., 2017).

STING-dependent DNA sensors include cGAS, DAIs, and IFI16. These DNA sensors play important roles in the defense against invasion of many DNA viruses, including HBV. A previous study showed that HBV genome-derived dsDNA induces the innate immune response through the cGAS-STING pathway and suppresses HBV assembly and replication (Dansako et al., 2016; He J. et al., 2016). Moreover, Liu et al. found MITA-related protein (MRP), a spliced isoform of MITA/STING, significantly inhibits HBV replication through the NF-κB pathway *in vitro* (Liu et al., 2017). Chen et al. also demonstrated that DAIs can inhibit HBV replication via activation of NF-κB but not IRF3 signaling in Huh7 cells (Chen et al., 2012). Recently, a study by Yang et al. (2020) showed that IFI16 serves as a unique nuclear sensor for HBV cccDNA and suppresses cccDNA function by integrating innate immune activation and epigenetic regulation by targeting the ISRE of cccDNA. However, higher HBsAg levels are associated with a stronger downregulation of innate immunity pathway regulators/effectors, including IFI16 (Lebossé et al., 2017). The DNA sensors mentioned above and their downstream adaptor protein, STING, have critical roles in the recognition and defense against HBV infection. However, CHB patients are unable to express appropriate levels of STING in PBMCs, which might result in impairing HBV DNA recognition, and subsequently, disruption of innate immune responses (Karimi-Googheri et al., 2015). This result is consistent with Kasap et al. (2020). Another study demonstrated that human and murine hepatocytes have limited expression of STING (Thomsen et al., 2016). The lack of a functional innate DNA-sensing pathway creates a suitable environment for HBV replication and contributes to the weak capacity to clear HBV infection. Likewise, a recent study by Lauterbach-Rivière et al. (2020) revealed that PHHs express reduced levels of DNA sensors as compared to myeloid cells, and the HBV DNA in the capsids is less likely to trigger the low functionality of the cGAS-STING pathway. These conditions likely allow HBV to escape the STING-dependent innate immune response.

## Hepatitis C Virus

Hepatitis C virus is a positive-strand RNA virus of the *Hepacivirus* genus, which belongs to the *Flaviviridae* family. HCV infection ultimately results in liver cirrhosis, hepatic failure, or HCC, all of which can cause death (Chevaliez and Pawlotsky, 2021). Numerous studies have shown that HCV is mainly sensed by RLRs, TLRs, and NLRs and has evolved routes to inactivate innate immune signaling.

### RIG-I-Like Receptors

**FIGURE 3:**
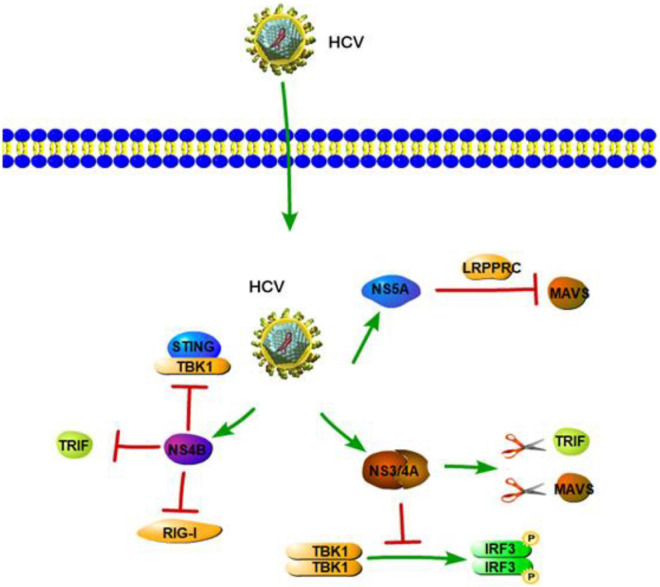
Hepatitis C virus suppression of type I interferon (IFN) response (green arrows indicate the related signaling pathways and T-arrows in red indicate blocking the targets). HCV NS3/4A serine protease cleaves TRIF and MAVS to inhibit IFN-b pathway. HCV NS3 protein suppresses IRF-3 activation through disrupting the association between TBK1 and IRF3. NS4B suppresses RIG-I-mediated IFN-β production signaling through a direct protein interaction with STING or disrupting the interaction between STING and TBK1.NS4B also could inhibit TLR3-mediated IFN signaling by downregulating TRIF protein levels via caspase 8. NS5A is able to bind with LRPPRC and exploit the ability of LRPPRC to inhibit MAVS-regulated antiviral signaling.

RIG-I-like receptors are pathogen receptors that regulate cellular permissiveness to HCV replication and play key roles against HCV infection. A previous study demonstrated that RIG-I can recognize the polyuridine motif of the HCV genome 3′ non-translated region (NTR) as a PAMP. The 3′ NTR of HCV stimulates RIG-I-dependent signaling to induce a hepatic innate immune response *in vivo* and triggers IFN and ISG expression to suppress HCV infection *in vitro* (Saito et al., 2008). Wu et al. also found two SNPs in RIG-I (*DDX58* rs10813831 and rs10738889) that are associated with spontaneous clearance of HCV in HCV-persistent infectors of the Chinese Han population. The two SNPs of RIG-I might be identified as a predictive marker in local HCV patients (Wu et al., 2019). Similar to HAV 3ABC, HCV NS3/4A can cleave MAVS at C508, releasing MAVS from the mitochondrial membrane and resulting in the subcellular redistribution of MAVS and loss of interaction with RIG-I (Figure 3; Loo et al., 2006). Moreover, HCV NS3/4A can also inactivate MAVS, which results in the downregulation of the RIG-I-dependent antiviral response, thereby allowing HCV to avoid triggering both IRF3 and NF-κB activation during infection (Foy et al., 2005; Meylan et al., 2005). A previous study revealed HCV NS3/4A negatively regulates other steps of the antiviral response as well, such as targeting the E3 ubiquitin ligase Riplet, which is known to activate RIG-I by triggering K63-linked polyubiquitination (Oshiumi et al., 2013). A further study by Vazquez et al. identified a Tyr-16-Phe (Y16F) change in the NS4A transmembrane domain that can prevent NS3-NS4A targeting of Riplet, but not MAVS. This result demonstrated that the NS4A Y16 residue regulates a non-canonical Riplet-TBK1-IRF3-dependent, but RIG-I-MAVS-independent signaling pathway, which limits HCV infection (Figure 3; Vazquez et al., 2019). In addition, Otsuka et al. (2005) found that HCV NS3 protein suppresses IRF-3 activation through disrupting the association between TBK1 and IRF3 (Figure 3). Like NS3/4A, the NS4B protein also blocks IFN signaling mediated by RIG-I. NS4B suppresses RIG-I-mediated IFN-β production signaling through a direct protein interaction with STING, and the disruption of this interaction might restore the cellular antiviral response (Figure 3; Nitta et al., 2013). Another mechanistic study indicated that NS4B can disrupt the interaction between STING and TBK1 to allow HCV to evade the host innate immune system (Ding et al., 2013). Another HCV protein, NS5A, in addition to its role in viral replication and assembly, is also able to bind with LRPPRC and exploit the ability of LRPPRC to inhibit MAVS-regulated antiviral signaling (Refolo et al., 2019). MDA5 is another RLR and plays an important role in sensing HCV infection to trigger IFN response (Israelow et al., 2014). Cao et al. (2015) demonstrated that MDA5 can recognize HCV to initiate the host innate immune response during HCV infection. They also considered that HCV infection is capable of inducing IFN production, which is mainly dependent upon MDA5 rather than RIG-I (Cao et al., 2015). At the same time, however, a study by Hiet et al. (2015) reported that HCV can activate both RIG-I- and MDA5-mediated signaling, and it is probably in a sequential manner, with MDA5-mediated signaling likely taking place during the later stages of the viral replication cycle as compared with RIG-I-mediated signaling. This suggested that RIG-I- and MDA5-mediated innate immune response may be connected with the progression of HCV infection. Such a complementary role of RIG-I and MDA5 has also been previously reported for another *Flaviviridae* family member, West Nile Virus (Fredericksen et al., 2008; Errett et al., 2013).

### Toll-Like Receptors and NOD-Like Receptors

As for TLRs and NLRs, a previous study revealed IL-1β production and hepatic inflammation in HCV infection are linked and driven via virus-induced TLR7 and NLRP3 inflammasome signaling in liver macrophages (Negash et al., 2013). Zhang et al. (2009) provided direct evidence that HCV RNA components can enhance immune activation via TLR7 in multiple types of cells, including PBMCs, pDCs, THP-1, and Huh7. Similar with HAV, Takahashi et al. (2010) showed that pDCs produce type I IFN by direct sensing of HCV-infected cells. Importantly, active HCV replication and TLR7 signaling are also required in this process (Takahashi et al., 2010). Although viral RNA is not a typical substrate of TLR9, it has been proposed that TLR9, a DNA-sensing TLR, takes part in HCV antiviral effects, such as activation of pDCs via TLR9 through the HCV RNA polyuridine tail (Howell et al., 2013). Zhou et al. (2009) demonstrated that HCV infection leads to a decreased expression of TLR9 mRNA and protein, which are negatively correlated with serum viral copies. Further, a clinical study showed that the TLR9 promoter SNPs are associated with the natural course of HCV infection and have higher transcriptional activities in women, suggesting that the DNA sensor TLR9 has an effect in HCV infection (Fischer et al., 2017). Moreover, TLR3-mediated chemokine and inflammatory cytokine response also plays a significant role in the host immune response to HCV. TLR3 can sense HCV dsRNA and induce NF-κB activation with the production of numerous chemokines and inflammatory cytokines (Li et al., 2012). The studies that HCV NS3/4A serine protease cleaves TRIF and also MAVS (Li et al., 2005a,b). NS3/4A-mediated cleavage of TRIF can inhibit the immune response induced by TLR3 (Li et al., 2005a). Moreover, NS4B, another HCV-encoded protein, also inhibits TLR3-mediated IFN signaling by downregulating TRIF protein levels via caspase 8 (Figure 3; Liang et al., 2018). Another clinical study showed that, as compared to healthy controls, the expression of TRIF, TLR3, and MAVS is downregulated in chronic hepatitis C (CHC) patients (Firdaus et al., 2014; Kar et al., 2017). According to the studies mentioned above, the ability of HCV NS3/4A/4B proteins to inhibit the innate immune occurs mainly via two ways. One is to downregulate crucial innate immune signaling pathway molecules, and the other is to inhibit the association between upstream and downstream signaling molecules. These two strategies prevent downstream activation of the immune response and help HCV to evade the host innate immune system.

Aside from inhibiting the innate immune response, viral proteins can also induce and maintain the innate immune response. HCV, through the action of its NS5A protein, induces expression of TLR4 in B cells and hepatocytes to improve the TLR4-mediated inflammatory response (Machida et al., 2006). The HCV core and NS3 proteins also trigger TLR2-specific cellular activation and induce inflammatory pathways in a TLR2-dependent manner, suggesting that the HCV core and NS3 protein are likely to contribute to inflammatory activation during HCV infection (Dolganiuc et al., 2004). Further study is needed to show that immune recognition mediated by TLR2 involves TLR1 and TLR6 (Loo et al., 2006).

## Hepatitis D Virus

Hepatitis D virus is the smallest known human virus with a circular RNA genome. As a satellite RNA virus, HDV does not encode its own envelope proteins for packaging of its ribonucleoprotein (RNP) and typically depends on the surface glycoproteins (GPs) of HBV for virion assembly, envelopment, and transmission (Hughes et al., 2011; Rizzetto, 2016). According to Giersch et al. (2015) significant induction of hISGs (hISG15, hSTATs, and hHLA-E) and human-specific cytokines (hIP10, hTGF-β, hIFN-β, and hIFN-γ) are detected in during HBV and HDV co-infection as compared with HBV mono-infection in humanized uPA/SCID/beige (USB) mice. Previous studies have also demonstrated that HDV can induce IFN-β and IFN-γ in hepatocytes and mouse models (Giersch et al., 2015; He et al., 2015; Alfaiate et al., 2016).

**FIGURE 4:**
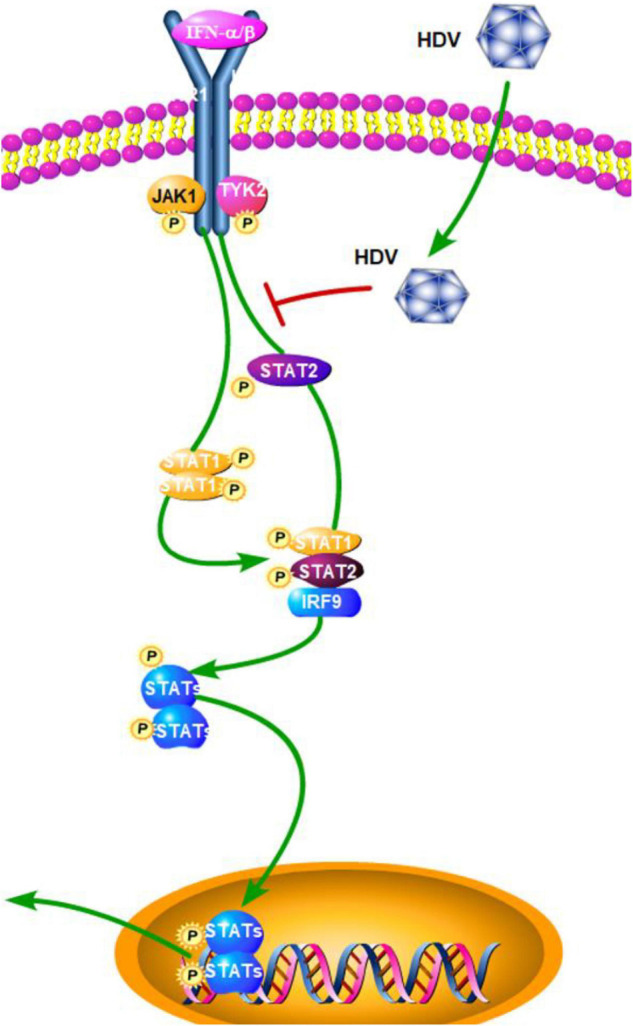
Hepatitis D virus suppression of type I interferon (IFN) response (green arrows indicate the related signaling pathways and T-arrows in red indicate blocking the targets). HDV impairs the IFN-α-stimulated JAK-STAT signaling pathway by inhibiting tyrosine phosphorylation of STAT1, STAT2, and receptor associated kinase Tyk2.

Although numerous molecules have been reported to participate in detecting intracellular viral RNA and triggering innate immune responses, to date it is not well-known which PRR(s) is/are involved in HDV infection and if this defense affects HDV replication. TLR3 uses TRIF as the main intermediate signaling molecule to induce IFN-β, while MDA5 and RIG-I use MAVS. In order to elucidate if one or both of the pathways is involved in the detection of HDV-RNA and IFN-β induction, Suárez-Amarán et al. (2017) established a new mouse model by using adeno-associated viruses (AAV; AAV-HDV and AAV-HBV) to deliver HDV- and HBV-replication competent genomes. This new HDV mouse model reproduces several important characteristics of human HDV infection and disease. The authors used TRIF- and MAVS-deficient mice to establish the HDV model. The results showed that in TRIF-deficient mice, IFN-β mRNA levels are equivalent to those observed in wild-type mice, while they are obviously reduced but not completely abolished in MAVS-deficient mice. A strong reduction in the expression level of all of the ISGs, except IP-10, was also observed in MAVS-deficient mice (Suárez-Amarán et al., 2017). This finding suggested that MAVS is the key adaptor protein and MDA5 or RIG-I, but not TLR3, might play a dominant part in the detection of cytoplasmic HDV dsRNA. Subsequently, a further study by Zhang et al. showed that HDV can induce IFN levels remained unaltered upon RIG-I or TLR3 knockdown, but are almost completely abolished upon MDA5 depletion. Although it has a minimal effect on HDV replication, MDA5 depletion greatly suppresses HDV-induced IFN response. These results revealed that MDA5 is the key sensor for the recognition of HDV-replicative intermediates rather than RIG-I and TLR3. According to Zhang et al. (2018) the precise mechanism of how HDV RNA is recognized by MDA5 still needs further research. In addition to this, a previous study by Pugnale et al. (2009) revealed that HDV impairs the IFN-α-stimulated JAK-STAT signaling pathway by inhibiting tyrosine phosphorylation of STAT1, STAT2, and receptor associated kinase Tyk2 (Figure 4). This result revealed a countermeasure for HDV to evade the IFN response by targeting the JAK-STAT transduction cascade. In general, the role of other PRRs and HDV viral factors in the innate immune system initiation and evasion still need further research. In order to achieve this, a more suitable HBV and HDV co-infection system should be established as soon as possible.

## Hepatitis E Virus

### The Recognition of Hepatitis E Virus

Aside from HAV, HEV is also a common cause of acute viral hepatitis in human. HEV is a non-enveloped virus with a positive-sense, single-stranded RNA genome that belongs to the family of *Hepeviridae* (Wang et al., 2016; Sander et al., 2018). During HEV infection, RLRs and TLRs mainly recognize HEV and trigger innate immune responses against HEV. A previous study revealed that a U-rich region in the 3′ UTR of the HEV genome mainly acts as a potent RIG-I PAMP, which induces an IFN response that is specific to the infected host cell type (Sooryanarain et al., 2020). Ectopic overexpression of RIG-I activates the transcription of many antiviral ISGs, partly through the activation of the JAK-STAT cascade without IFN production. In turn, the transcription of these antiviral ISGs helps establish an anti-HEV status (Xu et al., 2017). Unlike HAV and HCV, HEV does not target MAVS and can induce a sustained type III IFN response, which is dependent on both RIG-I and MDA5. However, the IFN level is insufficient to eliminate the virus (Yin et al., 2017). Li Y. et al. (2020) revealed that MDA5 can potently inhibit the replication of HEV without IFN production through the stimulation of a non-canonical IFN-like response, which is partially dependent on the JAK-STAT cascade.

### Hepatitis E Virus Proteins

**FIGURE 5:**
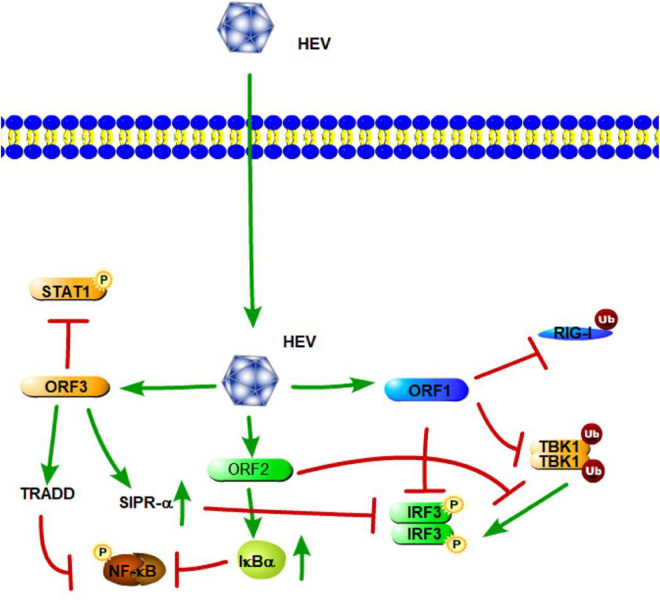
Hepatitis E virus suppression of type I interferon (IFN) response (green arrows indicate the related signaling pathways and T-arrows in red indicate blocking the targets). HEV ORF1 mediates the deubiquitination of RIG-I and TBK-1 and inhibits IRF-3 phosphorylation. The ORF2 protein affects RIG-I and TLR signaling pathways to block the phosphorylation of IRF3 by TBK1. The ORF2 protein also can stabilize inhibitor kappa B alpha (IκBα), thereby inhibiting NF-κB activity. HEV ORF3 contributes to the activation of SIRP-α to downregulate phosphorylation of IRF3. ORF3 of HEV suppresses TLR3-mediated NF-κB signaling via tumor necrosis factor receptor 1-associated death domain protein (TRADD). HEV ORF3 protein has the ability to inhibit IFN-α signaling through the regulation STAT1 phosphorylation.

Similar HEV sequences have been isolated from many different animals, and the HEV mainly contains open-reading frames (ORFs) 1, 2, and 3. According to previous studies, these ORFs encode HEV proteins, which both activate and suppress the innate immune response during HEV infection. HEV ORF1 encodes non-structural polyproteins, and some of them emerge as potential IFN antagonists. HEV methyltransferase (MeT), a non-structural protein of HEV, significantly inhibits RIG-I/MDA5-mediated IFN-β signaling and NF-κB activation in a dose-dependent manner, leading to attenuation of IFN-β production (Figure 5; Kang et al., 2018; Min and Min, 2019). This suppression of IFN-β signaling is mainly caused by inhibiting the phosphorylation and activation of IRF3 and the p65 subunit of NF-κB, thus blocking efficient activation of the IFN-β promoter (Myoung et al., 2019). Aside from Met, papain-like cysteine protease domain (PCP) and X domain, other products of HEV ORF1, have also been demonstrated to act as IFN antagonists, which can inhibit RIG-I-mediated signaling in different steps (Figure 5). PCP mediates the deubiquitination of RIG-I and TBK-1, while the X domain inhibits IRF-3 phosphorylation (Figure 5; Nan et al., 2014b). These are potential molecular mechanisms of HEV ORF1-mediated antagonism of IFN pathways.

ORF2 encodes the capsid protein of HEV. A previous study has shown that the capsid protein interacts with the MAVS-TBK1-IRF3 complex to block the phosphorylation of IRF3 (Figure 5). The arginine-rich motif of the N-terminal domain of the capsid protein has been found to be indispensable for inhibiting IRF3 activation (Nan et al., 2014a). Hingane et al. (2020) also indicated that the HEV ORF2 protein affects both the IFN-β and NF-κB axes of the RIG-I and TLR signaling pathways via targeting innate immune signaling downstream of adapter proteins and upstream of IRF3. Moreover, the ORF2 protein of HEV can stabilize inhibitor kappa B alpha (IκBα), thereby inhibiting NF-κB activity in human hepatoma cells (Figure 5; Surjit et al., 2012). Thus, HEV ORF2 protein most likely leads to a general suppression of the innate immune response.

HEV open-reading frame 3 (ORF3) encodes a 13-kDa multifunctional protein (vp13). A previous study showed that HEV vp13 can enhance poly (I:C)-induced IFN expression by increasing the RIG-I protein level. Additionally, vp13 interacts with the N-terminal domain of RIG-I and can enhance its K63-linked ubiquitination, which is essential for RIG-I activation (Figure 5; Nan et al., 2014a). Xu et al. (2014) also revealed that the ORF3 of HEV transiently activates NF-κB through an unfolded protein response (UPR) during the early stage of infection; but in the late phase, HEV ORF3 inhibits NF-κB signaling to create a favorable environment for HEV replication. During HEV infection, the D1 domain of HEV ORF3 contributes to the activation of SIRP-α, and thus, downregulates phosphorylation of IRF3, which results in the suppression of IFN-β (Huang et al., 2016). With regard to the TLRs involved in HEV, a study by Majumdar et al. (2015) revealed that TLR3 plays an important role in HEV disease pathogenesis. Patients expressing high levels of TLR3 are able to limit the disease and recover uneventfully (Majumdar et al., 2015). He M. et al. (2016) also demonstrated that the ORF3 of HEV downregulated TLR3-mediated NF-κB signaling via tumor necrosis factor receptor 1-associated death domain protein (TRADD) and receptor-interacting protein kinase 1 (RIP1) (Figure 5). Furthermore, Lei et al. (2018) found that HEV ORF3 protein significantly inhibits type I IFN induction by suppressing expression of TLR3/TLR7 and impairing multiple signaling pathways, including NF-κB, JAK/STAT, and JNK/MAPK. Aside from IFN-β signaling, a study by Dong et al. (2012) found that the HEV ORF3 protein has the ability to inhibit IFN-α signaling through the regulation STAT1 phosphorylation and reduced IFN-α-stimulated gene expression. Overall, these results showed how HEV triggers innate immunity through the direct or indirect inhibition of TLR/RLR-mediated activation of host innate immune responses.

## Conclusion

Many studies have documented how hepatitis viruses trigger and evade the host innate immune response. From these studies, it could conclude that host recognition of the various hepatitis viruses via PRRs is the first step to initiate the host innate immune response. TLRs and RLRs are the main PRRs that recognize hepatitis viruses. In addition, HBV dsDNA also can be recognized by DNA sensors. During infection, hepatitis viruses have evolved the ability to compromise the innate immune system. There are some similarities in immune evasion techniques between the different types of hepatitis viruses. Key signaling molecules, including PRRs and central adaptor proteins, play important roles in innate immune signaling pathways. Decreased expression of these key signaling molecules is an efficient and common strategy that hepatitis viruses use to escape immune supervision. The expression of several TLRs, RLRs, and adaptor proteins, such as TLR3, RIG-I, MAVS, and TRIF, decreased in some types of hepatitis virus infection. Hepatitis virus proteins or factors also have the capacity of interfering with the innate immune response, which aids in the evasion of the innate immune response. Some hepatitis viral proteins are involved in the cleavage and degradation of key signaling molecules, such as HAV 3ABC and HCV NS3/4A, which can cleave MAVS to disrupt host innate immune signaling pathways. Other hepatitis viral proteins directly or indirectly interfere with the function of key signaling molecules. For instance, HEV ORF3 can suppress innate immune signaling directly through TRADD and RIP1. It also activates SIRP-α and downregulates phosphorylation of IRF3 in an indirect manner. Moreover, some limiting factors still prevent the further research of hepatitis viruses. One limiting factor is the lack of suitable and well-defined small animal models, as it is important to validate the relationships between hepatitis viruses and host immunity under physiological conditions. With suitable animal models and systems construction, the relationship between hepatitis viruses and host innate immunity will be better understood, which will provide insights into anti-hepatitis virus therapy.

## Author Contributions

CX and JC wrote the manuscript. XC and JC revised the manuscript. All authors have read and agreed to the published version of the manuscript.

## Conflict of Interest

The authors declare that the research was conducted in the absence of any commercial or financial relationships that could be construed as a potential conflict of interest.

## Publisher’s Note

All claims expressed in this article are solely those of the authors and do not necessarily represent those of their affiliated organizations, or those of the publisher, the editors and the reviewers. Any product that may be evaluated in this article, or claim that may be made by its manufacturer, is not guaranteed or endorsed by the publisher.
